# Digital physical activity intervention via the Kidney BEAM platform in patients with polycystic kidney disease: a randomized controlled trial

**DOI:** 10.1093/ckj/sfaf041

**Published:** 2025-02-12

**Authors:** Juliet Briggs, Elizabeth Ralston, Thomas J Wilkinson, Christy Walklin, Emmanuel Mangahis, Hannah M L Young, Ellen M Castle, Roseanne E Billany, Elham Asgari, Sunil Bhandari, Kate Bramham, James O Burton, Jackie Campbell, Joseph Chilcot, Vashist Deelchand, Alexander Hamilton, Mark Jesky, Philip A Kalra, Kieran McCafferty, Andrew C Nixon, Zoe L Saynor, Maarten W Taal, James Tollitt, David C Wheeler, Jamie Macdonald, Sharlene A Greenwood

**Affiliations:** Renal Therapies Department, King's College Hospital, London, UK; Department of Women's and Children's Health, Faculty of Life Sciences, King's College London, London, UK; National Institute of Health Research Leicester Biomedical Research Centre, Leicester, UK; Renal Therapies Department, King's College Hospital, London, UK; Renal Therapies Department, King's College Hospital, London, UK; Leicester Diabetes Centre, University Hospitals of Leicester NHS Trust, Leicester, UK; Diabetes Research Centre, University of Leicester, Leicester, UK; Curtin School of Allied Health, Faculty of Health Sciences, Curtin University, Western Australia, Australia; Department of Cardiovascular Sciences, University of Leicester, Leicester, UK; Department of Nephrology, Guys and St Thomas's Hospital, London, UK; Department of Nephrology, Hull University Teaching Hospitals NHS Trust, Hull, UK; Centre for Nephrology, Urology and Transplantation, Faculty of Life Sciences, King's College London, London, UK; Department of Cardiovascular Sciences, University of Leicester, Leicester, UK; Faculty of Health, Education and Society, University of Northampton, Northampton, UK; Department of Psychology, Psychology & Neuroscience, King's College London, London, UK; Department of Nephrology Royal Free Hospital, London, UK; Department of Nephrology, Royal Devon and Exeter NHS Foundation Trust, Exeter, UK; Department of Nephrology, Nottingham NHS Trust, Nottingham, UK; Department of Nephrology Salford Royal Hospital, Northern Care Alliance NHS Foundation Trust, Salford, UK; Department of Nephrology, Barts Health NHS Trust, London, UK; Department of Renal Medicine, Lancashire Teaching Hospitals NHS Foundation Trust, Preston, UK; Division of Cardiovascular Sciences, The University of Manchester, Manchester, UK; School of Health Sciences, University of Southampton, Southampton, UK; Centre for Kidney Research and Innovation, School of Medicine, University of Nottingham, Nottingham, UK; Department of Nephrology Salford Royal Hospital, Northern Care Alliance NHS Foundation Trust, Salford, UK; Department of Renal Medicine, University College London, London, UK; Institute for Applied Human Physiology, Bangor University, Bangor, UK; Renal Therapies Department, King's College Hospital, London, UK; Centre for Nephrology, Urology and Transplantation, Faculty of Life Sciences, King's College London, London, UK

**Keywords:** digital health intervention, exercise, physical activity, polycystic kidney disease, quality of life

## Abstract

**Background:**

In people living with polycystic kidney disease (PKD), physical inactivity may contribute to poor health-related quality of life (HRQoL). To date, no research has elucidated the impact of a PKD-specific physical activity programme on HRQoL and physical health. This substudy of the Kidney BEAM Trial evaluated the impact of a PKD-specific 12-week educational and physical activity digital health intervention for people living with PKD.

**Methods:**

This study was a mixed-methods, single-blind, randomized waitlist-controlled trial. Sixty adults with a diagnosis of PKD were randomized 1:1 to the intervention or a waitlist control group. Primary outcome was difference in the Kidney Disease QoL Short Form 1.3 Mental Component Summary (KDQoL-SF1.3 MCS) between baseline and 12 weeks. Six participants completed individualized semi-structured interviews.

**Results:**

All 60 individuals (mean 53 years, 37% male) were included in the intention-to-treat analysis. At 12 weeks, there was a significant difference in mean adjusted change in KDQoL MCS score between the intervention group and waitlist control [4.2 (95% confidence interval 1.0–7.4) arbitrary units, *P =* .012]. Significant between-group differences in KDQoL subscales—burden of kidney disease (*P* = .034), emotional wellbeing (*P* = .001) and energy/fatigue (*P* = .001)—were also achieved. There was no significant between-group difference in KDQoL PCS scores (*P* = .505). Per-protocol analyses revealed significant between group differences in the PAM-13 patient activation score (*P* = .010) and body mass (*P* = .027). Mixed-methods analyses revealed key influences of the programme, including opportunities for peer support and to build on new skills and knowledge, as well as the empowerment and self-management.

**Conclusion:**

A PKD-specific digital health educational and physical activity intervention is acceptable and has the potential to improve HRQoL. Further research is needed to better understand how specific education and lifestyle management may help to support self-management behaviour.

## Graphical Abstract

**Graphical Abstract:**
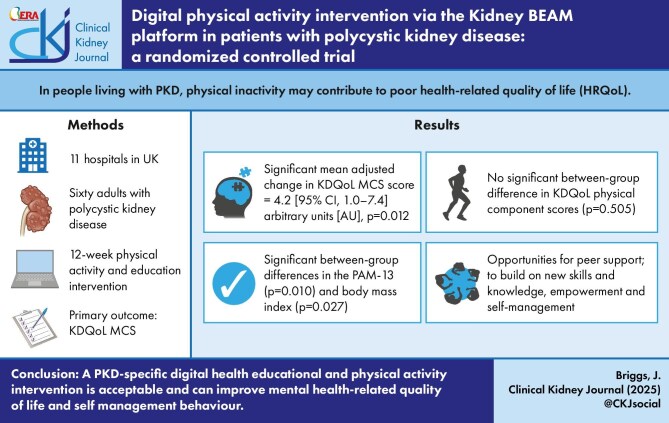

KEY LEARNING POINTS **What was known:**

- Mental health is detrimentally impacted for people with late-stage autosomal dominant polycystic kidney disease .
- Sedentary behaviour is high within the chronic kidney disease population.
- Despite recommendations for individuals living with polycystic kidney disease (PKD) to engage in physical activity interventions, limited research has been undertaken.

**This study adds:**

- A kidney-specific digital health physical activity platform, with specific PKD education, may improve an individual's health-related quality of life (HRQoL).
- Individuals valued a focus on health and well-being, particularly the opportunity for education, peer support and self-management.

**Potential impact:**

- The use of a PKD-specific education and physical activity digital health intervention may have the potential to support individuals living with the condition to improve their HRQoL and self-manage aspects of their condition.
- This specific digital health intervention may be of benefit as an adjunct to standard clinical management of people living with PKD.
- Further research is needed to focus on when this intervention could be offered to individuals living with PKD as part of their kidney care journey.

## INTRODUCTION

Autosomal dominant polycystic kidney disease (ADPKD) is the most commonly inherited type of kidney disease, affecting approximately 12.5 million people worldwide [1], and is responsible for up to 10% of end-stage kidney disease [2, 3]. Polycystic kidney disease (PKD) significantly affects psychological health and health-related quality of life (HRQoL), by causing pain, discomfort, fatigue, emotional distress and impaired mobility [4–6].

In recent years there have been advances in treatment approaches which have improved the HRQoL, as well as the lifespan, of these individuals. These approaches include early detection, lifestyle and weight management, hypertension optimization, and review of kidney and extra-kidney complications [2, 7]. Although no specific studies have investigated physical activity behaviours in people living with PKD, high levels of sedentariness are likely common, given similar patterns in individuals with chronic kidney disease (CKD) [8, 9]. As such, support to facilitate a physically active lifestyle could be beneficial for this population.

Despite the recommendations for people with PKD to take part in exercise-based rehabilitation, albeit with specific precautions to avoid high-impact sports due to the risks of cyst rupture [10], very little research has investigated the impact of physical activity and exercise interventions in people living with PKD [2]. Physical activity is likely to confer many physiological benefits, given that individuals with PKD have lower cardio-respiratory fitness compared with healthy controls [11, 12], along with a dysregulated cardiovascular response [10, 13] to exercise, reduced submaximal anaerobic threshold [11], diastolic dysfunction, raised sympathetic autonomous system response and early signs of arterial stiffness [10]. Early positive evidence in murine models with PKD has demonstrated that long-term exercise slows the progression of markers of PKD [14], but further research is needed to see if these observations translate to humans.

The use of digital health interventions (DHIs) as a vehicle to deliver lifestyle interventions for individuals with a range of health conditions, are gaining global popularity. In the UK, they are central to the National Health Service (NHS) Long Term Plan, which highlights the importance of DHIs to facilitate self-management of health and wellbeing needs in people living with long-term conditions, such as CKD [15]. A multicentre randomized controlled trial (RCT) recently showed that Kidney BEAM (www.kidneybeam.com), a kidney-specific DHI that delivers online lifestyle support interventions for individuals living with CKD, is an efficacious and cost-effective solution to improve mental HRQoL [16, 17]. This type of intervention is particularly important as, unlike other long-term conditions, currently there are very limited services that provide specific rehabilitation interventions for PKD [18] as part of routine clinical practice. This is despite best practice recommendations being in place [19]. The Kidney BEAM platform, which is now widely available for people living with CKD in the UK, is therefore an ideal place to create a bespoke education and physical activity training module to specifically support people living with PKD to engage in physical activity.

This study therefore aimed to understand whether a 12-week PKD-specific physical activity and educational DHI could effectively to improve HRQoL for people with PKD, and whether this would be an acceptable approach.

## MATERIALS AND METHODS

The main Kidney BEAM trial was a multicentre, single-blind, waitlist controlled trial designed to assess the effectiveness of a specific physical activity DHI on HRQoL in people living with CKD in the UK. Full trial design and protocol have been previously published [20]. The PKD Kidney Beam substudy was an exploratory pilot RCT that included 60 adults living with PKD, recruited in addition to participants within the main trial. The PKD substudy was approved by the Bromley NHS Research Ethics Committee (Ref: 21/LO/0243) and Health Research Authority, and was pre-registered on ClinicalTrials.gov (NCT04872933).

### Participants

Adults ≥18 years of age with a diagnosis of PKD were considered eligible. Individuals needed to be able to access a DHI, using a digital device and WiFi connectivity, to be considered for the trial. Participants were recruited from 11 UK kidney centres. Potential participants were screened by their clinical team, and recent clinical records were reviewed to confirm eligibility at the time of enrolment. Suitable adults were approached in person during routine clinic visits, or via telephone, by trained research staff. A complete list of the inclusion and exclusion criteria has been published previously [20]. All participants provided fully informed written consent. Eight individuals in the intervention group were later purposively sampled and invited to participate in a semi-structured telephone interview, to explore their experiences and views around the acceptability of the PKD-specific content on the Kidney BEAM platform.

### Randomization

Participants were randomly assigned 1:1 to the Kidney BEAM intervention group or the waiting list control group (usual care). Randomization was performed by an independent member of the research team using the Sealed Envelope web-based system. Due to the nature of the intervention, it was not possible to blind either the healthcare professionals delivering the physical activity intervention, or participants.

### Procedures

The Kidney BEAM trial intervention has been described in detail previously [20]. The PKD-Kidney Beam substudy participants who were randomized to the intervention group were directed to complete a PKD-specific education module, before starting the 12-week physical activity training programme, as per protocol [20]. The education module provided participants with tailored information around the importance of keeping active whilst living with PKD, as well as specific physical activity guidance and advice around how to keep active, and what physical activity is beneficial for this population. This was delivered by a specialist kidney exercise physiologist. The physical activity training has been described elsewhere [20]. Briefly, it consisted of two physical activity sessions per week, including a graded warm-up and cool-down. Structured aerobic and strength training exercises were led by specialist kidney physiotherapists, and involved two practitioners, one demonstrating the movements in standing and one demonstrating the movements seated in a chair. Participants completed baseline and 12-week assessments, as per protocol, and were invited to feedback on the acceptability of the module via one-to-one semi-structured telephone interviews. Interviews were conducted between September and October 2023 by one of the research team independent from the quantitative data collection.

### Outcomes

The primary outcome for this exploratory substudy was the between-group difference in Kidney Disease QoL Short Form 1.3 Mental Component Summary (KDQoL-SF1.3 MCS) at 12 weeks.

Secondary outcomes included the between-group difference in the Kidney Disease QoL Short Form version 1.3 Physical Component Score (KDQoL-SF1.3 PCS) and other subscales—patient activation (Patient Activation Measure-13, PAM-13), the European Quality of Life 5 dimension 3 level (EQ-5D-3L) utility score, physical function via the 60-s sit-to-stand test (STS-60), body mass index (BMI), haemoglobin and estimated glomerular filtration rate (eGFR— at 12 weeks, and a qualitative exploration of participant experiences of the intervention and trial procedures. Participants for the qualitative study were purposively sampled to ensure there was good representation in terms of age, sex, gender and ethnicity. All outcome measures chosen are known valid and reliable tools to measure the primary and secondary outcomes in CKD [21, 22], and all questionnaires were completed online. The STS-60 test was completed at home and observed via video conference by a research assistant.

### Statistical analysis

#### Quantitative analysis

In this *a priori* planned substudy, by design the study was not powered to detect statistical differences between intervention and control groups. Statistical analysis followed the same as the main Kidney BEAM trial intervention [16]. Exploratory analyses, with the presentation of confidence intervals, were used to explore differences between groups and answer the primary question: whether people living with PKD respond positively to the Kidney BEAM intervention, as was shown previously in the main study/complete sample. Primary and secondary outcomes were analysed with an analysis of covariance model, with baseline data and age as covariates. Independence of covariates, and approximated normality of residuals, were confirmed for all analyses. Quantitative analyses were performed in the intention-to-treat (ITT) population, using a last observation carried forward (LOCF) approach to missing data, which gives the most conservative result. Per-protocol (PP) analyses were also completed, in which only cases with observations at both baseline and Week 12 were included. Two-sided *P*-values of <.05 were considered to indicate statistical significance. Analyses were performed using IBM SPSS (version 28).

#### Qualitative analysis

Interviews were audio recorded, manually transcribed verbatim (E.R.), and subsequently analysed using an inductive thematic analysis approach (E.R.) [23]. Qualitative data were managed using Nvivo V14 (version 14.23), The coding and themes were reviewed independently by an author not involved in the coding process (J.B.), to check for suitability on two randomly selected transcripts. Reporting of qualitative data is informed by the qualitative research reporting guidelines (COREQ) [24]. Please see Supplementary material 5.0.

#### Mixed-methods analyses

Quantitative and qualitative data collection and analyses occurred concurrently, and independently before being analysed and combined. Results are discussed together in a ‘joint display’ to facilitate an overall assessment of acceptability (Supplementary Material 3.0).

## RESULTS

### Participant characteristics

**Figure 1:**
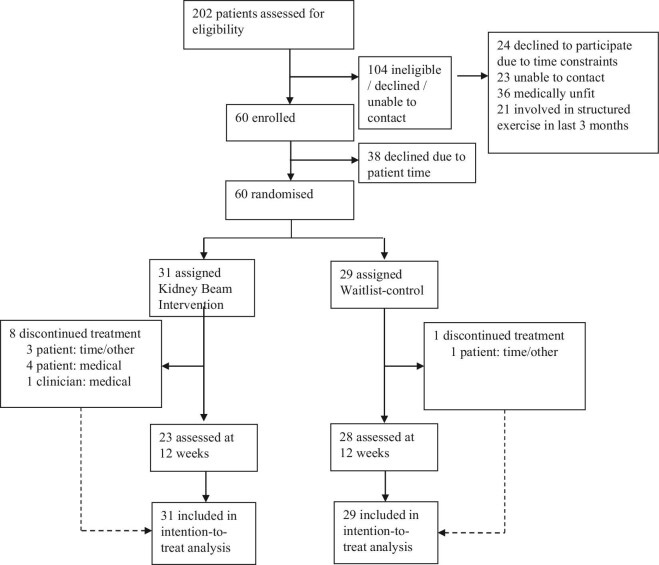
Trial profile.

Sixty individuals participated, 31 in the intervention group and 29 in the waitlist control group (see Fig. 1). Six out of eight individuals approached via telephone for an interview participated. Baseline characteristics of the total cohort are presented in Table 1.

**Table 1:** Baseline characteristics.

	Total (*n* = 60)	Intervention group (*n* = 31)	Waitlist control group (*n* = 29)
Age (years, SD)	53.2 (11.8)	53.2 (11.8)	50.3 (11.2)
Sex (*n*, %)			
Female		19 (61)	19 (65)
Male		12 (39)	10 (35)
Ethnicity (*n*, %)			
Black	2 (3)	0 (0)	2 (7)
White	50 (83)	27 (87)	23 (79)
Asian	7 (12)	4 (13)	3 (10)
Biracial	1 (2)	0 (0)	1 (3)
BMI (kg/m^2^)			
Median		26.3	29.2
IQR		23.9–31.0	24.0–32.7
Smoking (*n*, %)			
Current	1 (2)	1 (3)	0 (0)
Former	19 (32)	12 (39)	7 (24)
Never	40 (67)	18 (58)	22 (76)
Alcohol (*n*, %)			
More than recommended	3 (5)	3 (10)	0 (0)
Less than recommended	31 (52)	24 (77)	7 (24)
Non-drinker	26 (43)	4 (13)	22 (76)
Blood pressure (mmHg)			
Systolic blood pressure		135.4 (17.0)	133.9 (16.5)
Diastolic blood pressure		81.8 (10.1)	82.8 (7.2)
Resting heart rate (bpm)			
Mean		75.5	72.9
SD		16.7	13.3
Comorbidities (*n*, %)			
Cerebrovascular accident	2 (3)	2 (7)	0 (0)
Myocardial infarction	0 (0)	0 (0)	0 (0)
Diabetes mellitus	6 (10)	3 (10)	3 (10)
Hypertension	45 (75)	25 (81)	20 (69)
CKD stage (*n*, %)			
2	14 (23)	6 (19)	8 (28)
3a	13 (22)	4 (13)	9 (31)
3b	15 (25)	8 (26)	7 (24)
4	9 (15)	7 (23)	2 (7)
5	9 (15)	6 (19)	3 (10)
Treatment modality (*n*, %)			
Non-dialysis-dependent kidney disease	39 (65)	19 (61)	20 (69)
Kidney transplant recipient	16 (27)	8 (26)	8 (26)
Dialysis therapy	5 (8)	4 (13)	1 (3)
HbA1c (mmol/mol)			
Median		33.5	36
IQR		33–39	35–43
Hb (g/L)			
Mean		130.7	129.3
SD		15.3	20.2
eGFR (mL/min)			
Median		33.5	50.0
IQR		19.2–52.2	30.0–60.0
CRP (mg/L)			
Median		9.0	2.7
IQR		1.1–16.3	1.1–6.5
STS-60 (reps)			
Median		25	25
IQR		20–32	19–31

Hb, haemoglobin; SD, standard deviation.

Overall, the groups were well-balanced at baseline (see Table 1), with a higher proportion of females in both (63%). A higher proportion of individuals had non-dialysis dependent PKD (65%), in comparison with kidney transplant (27%) and dialysis therapy (8%). Baseline creatinine and C-reactive protein (CRP) were higher in the intervention group (creatinine 152 µmol/L, CRP 9.0 mg/L), compared with the usual care group (creatinine 100 µmol/L, CRP 2.7 mg/L). There were no significant differences in characteristics for those participants who completed, or did not complete, the intervention. A median of 14 [interquartile range (IQR) 6–20] of the recommended 24 sessions of structured physical activity were completed by participants in the Kidney BEAM intervention group, representing a median adherence rate of 67% (IQR 46–117). Participants completed a median of 481 min (IQR 156–945) of structured physical activity (on-platform and off-platform), which is the equivalent of 40 min per week. A median of 7 (IQR 2–11) of the recommended 12 sessions of education were completed, representing a median adherence rate of 58% (IQR 13–92).

Participants invited to qualitative interviews were purposively sampled. Baseline characteristics are presented in Table 2.

**Table 2:** Baseline characteristics of qualitative interview participants.

Sex	Age (years)	Ethnicity	eGFR (mL/min/1.73 m^2^)^a^	CKD stage	Unit	No. of physical activity sessions completed	No. of PKD education sessions completed
Male	63	White	24	4	Tx	11	3
Female	63	White	10	5	CKD	1	2
Male	45	White	59	3	CKD	9	3
Female	67	Other	34	3	CKD	10	3
Female	43	Other	>90	1	CKD	3	3
Male	42	White	45	3	CKD	6	3

a Calculated using Chronic Kidney Disease Epidemiology Collaboration 2009 equation without ethnicity adjustment.

Tx, transplant.

### Primary outcome—KDQoL-SF1.3 MCS

Table 3 and the Supplementary Material 1.0 present changes in HRQoL, as measured by the KDQoL-SF1.3 MCS. ITT analyses (Table 3) revealed statistically significant between-group differences in mental HRQoL at 12 weeks [+4.2 (95% confidence interval 1.0 to 7.4), *P* = .012]. This was also reflected in the PP analysis (Supplementary material 1.0), which demonstrated a statistically significant difference in the KDQoL-SF1.3 MCS score [+4.2 (95% confidence interval 1.0 to 7.4), *P* = .001].

**Table 3:** ITT analysis results (LOCF).

		Baseline	12 weeks	Mean difference in change between groups (Kidney BEAM—waitlist control)	
Outcome measure	*n*	Mean (SD)	Mean (SD)	Mean (95% CI)	*P-*value
Primary outcome					
KDQoL MCS (AU)					
Kidney BEAM	31	45.4 (10.2)	49.1 (10.7)	4.2 (1.0 to 7.4)	0.012
Waitlist control	29	47.9 (10.0)	46.4 (9.3)		
Secondary outcomes					
KDQoL PCS (AU)					
Kidney BEAM	31	44.6 (10.9)	44.1 (11.0)	–1.2 (–4.8 to 2.4)	0.505
Waitlist control	29	43.7 (11.9)	44.7 (13.1)		
Symptom problem list					
Kidney BEAM	26	83.2 (12.3)	83.7 (12.1)	–0.8 (–4.5 to 2.9)	0.676
Waitlist control	28	81.6 (17.2)	82.9 (16.7)		
Effects of kidney disease					
Kidney BEAM	31	77.8 (20.6)	74.7 (22.5)	–2.1 (–12.1 to 7.9)	0.673
Waitlist control	29	83.9 (15.5)	81.9 (22.7)		
Burden of kidney disease					
Kidney BEAM	31	62.5 (30.3)	68.7 (26.7)	8.0 (0.6 to 15.3)	0.034
Waitlist control	29	73.7 (23.4)	70.3 (26.2)		
Work status					
Kidney BEAM	31	75.0 (38.1)	75.0 (35.9)	4.3 (–15.3 to 6.6)	0.43
Waitlist control	29	81.0 (33.8)	84.5 (33.0)		
Cognitive function					
Kidney BEAM	31	78.1 (16.1)	84.4 (12.4)	5.4 (–0.6 to 11.4)	0.078
Waitlist control	29	78.6 (17.6)	79.8 (21.4)		
Quality of social interaction					
Kidney BEAM	31	75.2 (17.4)	77.1 (14.8)	–1.3 (–8.2 to 5.6)	0.701
Waitlist control	29	73.8 (15.9)	77.7 (19.5)		
Sexual function					
Kidney BEAM	15	28.3 (41.0)	30.6 (42.5)	–15.3 (–48.2 to 17.6)	0.348
Waitlist control	14	48.2 (46.5)	57.1 (46.7)		
Sleep					
Kidney BEAM	31	55.7 (17.2)	58.2 (19.0)	0.3 (–6.9 to 7.5)	0.934
Waitlist control	29	64.2 (17.1)	65.4 (20.8)		
Social support					
Kidney BEAM	30	77.4 (30.9)	83.8 (22.6)	9.5 (–2.9 to 22.0)	0.129
Waitlist control	25	79.3 (24.2)	79.5 (30.6)		
Dialysis staff encouragement					
Kidney BEAM	9	86.1 (22.0)	86.1 (22.0)	–2.3 (–9.7 to 5.0)	0.506
Waitlist control	7	75.0 (23.9)	72.2 (22.3)		
Overall health					
Kidney BEAM	31	61.2 (19.1)	62.8 (18.5)	–4.3 (–11.1 to 2.4)	0.201
Waitlist control	29	61.7 (20.0)	67.6 (21.5)		
Patient satisfaction					
Kidney BEAM	12	80.5 (12.0)	76.7 (25.0)	1.5 (–9.0 to 12.1)	0.766
Waitlist control	11	78.8 (21.2)	76.2 (21.4)		
Physical functioning					
Kidney BEAM	31	74.1 (21.7)	73.6 (22.4)	–0.6 (–10.2 to 9.0)	0.904
Waitlist control	29	76.5 (28.8)	75.7 (28.2)		
Role physical					
Kidney BEAM	31	67.2 (35.6)	68.7 (35.9)	2.5 (–14.8 to 19.7)	0.776
Waitlist control	29	63.8 (43.6)	65.5 (45.0)		
Pain					
Kidney BEAM	31	67.3 (26.4)	66.2 (23.7)	–3.6 (–13.4 to 6.1)	0.458
Waitlist control	29	63.7 (29.1)	67.2 (33.2)		
General health					
Kidney BEAM	31	43.9 (21.7)	43.1 (22.3)	–4.1 (–10.5 to 2.3)	0.208
Waitlist control	29	42.6 (21.2)	45.7 (23.4)		
Emotional wellbeing					
Kidney BEAM	31	70.1 (17.2)	76.2 (15.8)	9.5 (3.9 to 15.1)	0.001
Waitlist control	29	73.0 (17.5)	69.1 (18.2)		
Role emotional					
Kidney BEAM	31	71.9 (40.7)	71.9 (41.6)	–8.9 (–24.1 to 6.3)	0.245
Waitlist control	29	75.9 (38.7)	81.6 (32.8)		
Social function					
Kidney BEAM	31	69.1 (23.5)	75.4 (24.1)	2.8 (–6.5 to 12.0)	0.552
Waitlist control	29	64.6 (34.9)	69.0 (32.0)		
Energy/fatigue					
Kidney BEAM	31	43.3 (22.9)	52.5 (23.2)	8.9 (2.1 to 15.7)	0.011
Waitlist control	29	44.0 (24.4)	44.1 (24.7)		
EQ-5D-3L utility score					
Kidney BEAM	31	0.75 (0.18)	0.74 (0.21)	–0.03 (–0.09 to 0.04)	0.428
Waitlist control	29	0.76 (0.26)	0.77 (0.20)		
CFS					
Kidney BEAM	31	2.32 (0.83)	2.13 (0.76)	0.14 (–0.15 to 0.44)	0.337
Waitlist control	29	2.38 (0.82)	2.03 (0.78)		
STS-60					
Kidney BEAM	31	25.45 (8.21)	26.61 (9.47)	–1.37 (–3.60 to 0.86)	0.223
Waitlist Control	29	25.69 (8.61)	28.17 (10.62)		
PAM-13					
Kidney BEAM	31	62.36 (17.28)	68.43 (17.09)	7.4 (1.3 to 13.5)	0.018
Waitlist control	29	68.90 (16.97)	66.06 (17.63)		
PHQ-4					
Kidney BEAM	31	2.78 (3.65)	2.56 (3.45)	–0.03 (–1.2 to 1.1)	0.951
Waitlist control	29	2.14 (2.95)	2.24 (3.15)		
WSAS					
Kidney BEAM	30	9.10 (8.77)	8.87 (8.92)	–0.03 (–2.9 to 2.8)	0.982
Waitlist control	29	9.55 (10.74)	9.28 (10.60)		
eGFR					
Kidney BEAM	30	37.50 (25.10)	37.33 (25.27)	2.1 (0.3 to 4.0)	0.026
Waitlist control	23	46.74 (24.07)	44.17 (22.75)		
Hb					
Kidney BEAM	27	130.70 (15.26)	126.70 (20.61)	–3.2 (–11.1 to 4.6)	0.410
Waitlist control	29	129.34 (20.19)	129.17 (18.74)		
BMI					
Kidney BEAM	31	82.87 (17.94)	83.75 (18.24)	1.0 (–0.01 to 1.9)	.052
Waitlist control	28	82.10 (16.30)	82.02 (16.54)		

LOCF: last observation carried forward approach; SD, standard deviation; CI, confidence interval; AU: arbitrary units; CFS: Chalder Fatigue Score; Hb: haemoglobin.

### Secondary outcomes

Secondary outcomes, as measured through the KDQoL PCS, did not show significant significance in ITT (*P* = .505) or PP analysis (*P* = .621). However, several of the subscale components were significantly improved in the ITT results, including the burden of kidney disease (*P* = .034), emotional wellbeing (*P* = .001) and energy/fatigue (*P* = .001). These results were also demonstrated in the PP analyses, where the burden of kidney disease (*P* = .019), emotional wellbeing (*P* = .001), energy/fatigue (*P* = .004) and cognitive function (*P* = .022) were significantly improved at 12 weeks.

The EQ-5D-3L utility score was not significantly different between groups at 12 weeks (*P* = .428) in either the ITT or PP analyses. PP analyses demonstrated a statistically significant improvement in the PAM-13 patient activation score (*P* = .010), eGFR (*P* = .043) and BMI (*P* = .027). The Work and Social Adjustment Scale (WSAS) (*P* = .691) and the anxiety and depression questionnaire, as measured by the Physical Health Questionnaire (PHQ)-4 (*P* = .622), were not significantly changed by the intervention. For full results please see Table 3 and Supplementary material 1.0.

Two serious adverse events were recorded during the trial, both of which were unrelated to the study treatment. No expected related or unrelated serious adverse events were recorded in either group during the trial period (see Table 4).

**Table 4:** Number of patients with at least one serious adverse event during the Kidney BEAM trial by MedDRA system organ class.

	Total (*n* = 60)	Kidney BEAM group (*n* = 31)	Waitlist control group (*n* = 29)
Number of patients with any event	2 (3)	1 (2)	1 (2)
Surgical and medical procedures	1 (2)	0	1 (2)
Infections and infestations	1 (2)	1 (2)	0

Data are *n* (%).

MedDRA, Medical Dictionary for Regulatory Activities.

### Qualitative results

The analyses identified three key themes, with two associated sub-themes—Theme 1: individualized acceptance; Theme 2: influences of engagement; and Theme 3: complementary empowerment.

#### Theme 1. Individualized acceptance

Individual differences appeared to influence individuals’ acceptance and experience of interacting with PKD-Kidney Beam. This includes the attitudes and emotions they have associated with having PKD, as well as their experience of PKD.

*1.1. Individual attitudes*

Individuals with PKD demonstrated a range of complex emotions, including guilt, a sense of being fortunate in comparison with other people living with PKD, and feelings of unfairness. These emotions were often present in the context of comparing their experiences with others, especially those of family members with PKD. Negative emotions occasionally fostered the desire to avoid the reality of PKD and thus regarded Kidney BEAM as an unwelcome reminder. Whereas others reported positive attitudes towards Kidney BEAM, believing it has the potential to enhance their QoL and improve their PKD experience. For these participants, Kidney BEAM represents a source of hope amidst their struggles with PKD.

(In a discussion about the participant's brother) “Sadly, he's further down the line in terms of his kidney function, his has taken a rather, you know, serious nosedive and he's heading for dialysis now – even though he's 6 years younger than me, so you can't imagine how I feel about that”

(KB387)

*1.2. Identity and symptom experience*

Individuals’ personal experience of their PKD, and how they identify their kidney disease, influenced how accepting they were of PKD-Kidney Beam. Those who identified themselves as having PKD, as opposed to CKD generally, were typically more accepting. Conversely, one individual, who had undergone a kidney transplant, found PKD-Kidney Beam less appropriate, as they no longer identified as having PKD; rather considering themselves a transplant patient.

“I might not be the ideal subject really for this, cause I never thought of it as polycystic kidney disease, I just thought, the kidney was failing you know”

(KB356)

#### Theme 2. Influences of engagement

This theme incorporates how factors, such as the sense of community, the timing of when PKD-Kidney Beam is offered, and an individual's PKD severity, can shape people's experiences and acceptance of PKD-Kidney Beam.

*2.1. PKD community*

Individuals conveyed a desire for a PKD community, and PKD-Kidney Beam contributed towards this need, by offering live sessions which fostered a sense of belonging and personal connection, increased engagement, and increased accountability. The educational sessions were regarded as informative and beneficial, but individuals indicated they may not re-engage with them due to the content not changing. Participants valued a sense of community—to share advice, normalize medication side effects and exchange lifestyle tips.

“It's that connection with people in the same position and that there's something you can join that sort of thing”

(KB378)

*2.2. Severity and timing*

Individuals reported PKD-Kidney Beam to be both an informative and reassuring platform. Although several individuals wished they could have had access to PKD-Kidney Beam at the time of their initial diagnosis, to help them better understand PKD and anticipate their journey, some of those in earlier stages found PKD-Kidney Beam to be less relevant to them and perceived it to be more suitable for those with severe cases.

“I was surprised there was all that information out there actually. I wish I had that from day 1 when I was diagnosed, it would have been so helpful” (KB387)

#### Theme 3. Complementary empowerment

It is apparent that PKD Kidney Beam complements individuals’ clinical care they receive, through having a resource that enhances knowledge and enables people to have more reassurance and confidence with their PKD.

*3.1. Filling in the gaps*

Participants described being under a kidney clinical care team, which they occasionally have brief face-to-face contact with. PKD-Kidney Beam helped to maintain their care during the gap between consultations, complementing their medical care. This was most notably through addressing knowledge gaps, which was often attributed to the limited contact time between individuals and their healthcare professionals. The educational diagrams and videos within PKD-Kidney Beam were reported to significantly improve understanding and were positively embraced. Individual's clinical care team endorsed and provided PKD-Kidney Beam which enhanced initial engagement and fostered trust.

“Yeah there was one in particular that explained the disease quite well. It explained a bit about the… I think it was explained better to me in the video than it was by my consultant if I'm honest” (KB381)

*3.2. Empowerment*

Most participants reported that PKD-Kidney Beam offers an accessible and adaptable platform, which helps to empower them within their own PKD journey. Individuals found that easy access to the platform enabled them to engage flexibly, adapting to busy periods while maintaining continuous availability. The PKD specificity of the content encouraged individuals’ motivation and provided reassurance. Enabling and embracing individuals to have a proactive role in their PKD journey was regarded as a positive transformation.

“Yeah, I felt good. I felt like I had some exercise which is great. I felt motivated” (KB388)

The themes generated from this analysis suggest that PKD-Kidney Beam is a platform accepted and valued by PKD individuals.

“…So presumably, you find you've got polycystic kidneys are they now going to introduce you to kidney beam as a matter of course? Cause I think they should” (KB387)

#### Mixed-methods analysis

The integrated qualitative and quantitative findings allowed for further exploration of the results described from the quantitative analysis regarding; KDQOL-MCS, KDQOL-PCS and relevant subscales, PHQ-4 and PAM-13 outcome measures. These results suggest positive agreement regarding HRQoL, and patient activation, in response to 12 weeks of access to PKD-Kidney Beam. There was partial discord in anxiety and depression scores between quantitative and qualitative results, with interviewees reporting improvement in anxiety and depression despite this not being reflected in the quantitative results. Table 5 combines the qualitative and quantitative results in a joint display table.

**Table 5:** Joint display depicting mixed-methods results.

Concept being assessed	Quantitative results	Qualitative theme	Qualitative results and meaning	Mixed methods inferences
Health-related quality of life and sub scales	KDQOL MCS (ITT analysis) (*P* = .012)	Theme 1: individual attitudes Theme 2: influences to engagement	Some individuals expressed a feeling of guilt and unfairness with being diagnosed with PKD however, the specific PKD content on Kidney Beam offered a sense of hope and community engagement	Complimentary
	KDQOL PCS (PP analysis) (*P* = .621)	Not discussed as main objective of qualitative interviews however, discussed in main Kidney BEAM trial		Silence
	Burden of kidney disease (*P* = .019)	Theme 1.0: individualized acceptanceSub-theme 1.2: identity and experience	Individuals reported a range of emotions and feelings around their PKD diagnosis. This largely was influenced by their stage of disease	Partially complimentary
		Theme 2.0: influences to engagementSub-theme 2.1: PKD communitySub-theme 2.3: severity and timing	They felt that utilising a specific platform enabled them to gain peer support which was valued in helping to live with their condition and links to improvement in perceived burden reflected in quantitative results	
			Individuals reported the importance of the timing of offering of this resource dependent on the stage of their disease and therefore identity with PKD	
	Cognitive function (*P* = .022)	N/A	No discussion	Silence
	Emotional wellbeing (*P* = .001)	Theme 2.0: influences to engagement Theme 3.0: complimentary empowerment	PKD-Kidney BEAM provided individuals with a sense of community, motivation and engagement which was deemed to be valuable in managing their own condition This links well to the positive outcome seen with emotional wellbeing in the quantitative data	Complimentary
Anxiety and depression	PHQ-4 (*P* = .622)	Theme 1.0: individual attitudes Sub-theme 2.1: identity and symptom experience	Individuals discussed their identity and experience of being diagnosed with PKD, alongside potential for guilt with this being an inherited disease Identity and experience varied amongst interviewees dependent on their stage of disease. This linked to their sense of wellbeing and in some instances feelings of ‘being a fraud’ in their perceived health in comparison to others	Partial Discord
Patient activation (knowledge skills and confidence)	PAM-13 (PP analysis) (*P* = .010)	Theme 2.0: Influences to engagement Sub-theme 2.1: PKD community Theme 3.0: complementary empowerment	Individuals expressed the positive impact of being able to engage and connect with other individuals in the same position as them, and this influenced motivation to engage with the platform The educational content provided further insight, and positive re-enforcement in self-management of PKD	Complimentary

## DISCUSSION

This study aimed to evaluate whether a 12-week PKD-specific educational and physical activity DHI programme (PKD Kidney Beam) could improve mental HRQoL for people living with PKD. The results revealed a significant improvement in the KDQoL MCS, suggesting that this PKD-specific DHI has the potential to improve mental HRQOL for people with this inherited condition. Mixed-methods analyses of the data revealed several key influences of the PKD Kidney BEAM programme that contributed to improvements in mental and physical wellbeing. These included the opportunity for peer support and a sense of community, particularly for individuals who struggled with a sense of identity and guilt about their PKD diagnosis, the opportunity to build on new skills and knowledge, as well as the empowerment and self-management of their condition. To our knowledge, this is the first study to investigate the use of a DHI to deliver a PKD-specific physical activity and education programme. These results echo those of the larger Kidney BEAM trial [16] in a broad population of people living with CKD.

PKD often poses a significant symptom burden for individuals living with the condition [25, 26], significantly impacting upon their QoL [25, 27]. This has an impact with regards to pain management, fatigue and ability to carry out daily activities [25, 28, 29]. Impairments in work productivity and daily activities have shown to be impacted both in early and later stages of disease [29]. Promisingly, secondary outcomes including the burden of kidney disease, emotional wellbeing and energy/fatigue, were improved by the PKD Kidney Beam DHI in this study. This demonstrates the benefit of this type of intervention and the potential to enable individuals to self-manage some of the symptoms experienced.

The psychological impact of PKD has been investigated in recent research [4]. This includes the burden of knowledge of the disease process, as well as the psychological impact of PKD being an inherited disease, and often individuals having witnessed other relatives going through treatment for PKD, which may result in significant psychological impact [30]. ADPKD presents with a number of physical symptoms, which may also influence an individual's overall QoL. These may include chronic pain, hypertension, the development of cysts in other organs and gastrointestinal complications [30]. The observed significant improvement in HRQoL in this substudy therefore indicates a clinically meaningful benefit for people living with PKD. Qualitative analysis revealed the mental health impact of living with PKD, and the potential of this intervention to support a sense of community, as well as to empower individuals, and facilitate self-management of their condition. Although the PHQ-4 was not shown to be significant, qualitative analysis revealed the impact of living with PKD on mental health, including influencing their sense of identity. However, importantly the use of PKD-Kidney Beam appeared to build a sense of community, and facilitate peer support, which positively influenced emotional wellbeing and provided the opportunity to engage with others living with the same condition.

Self-management is gaining increasing importance in healthcare settings, particularly in relation to managing long-term health conditions, such as CKD [31]. Self-management refers to an individual taking an active role in their health and management of their condition [32]. To achieve this, individuals are required to achieve a term labelled ‘patient activation’. This involves an individual having the knowledge, skills and confidence needed to perform the desired behaviours to manage their own health [32]. It is therefore promising that the results from this PKD substudy revealed significant improvements in patient activation, highlighting its potential in individual lifestyle self-management for people with PKD.

Whilst there were significant improvements in primary and secondary outcomes achieved in this current PKD substudy that are comparable to the main Kidney BEAM trial [16], a notable contrast in the qualitative results from this PKD substudy were revealed to be around individual attitudes. Qualitative analyses revealed that those individuals with PKD reported complex emotions, including guilt, particularly if other family members were also diagnosed with this inherited disease, a feeling of in some instances of unfairness or of feeling fortunate in comparison with others. This is echoed in other literature, where counselling to reduce the burden of ‘genetic guilt’ was seen as an important aspect of care [25]. Additionally, whilst the PKD-specific DHI was welcomed by some people with PKD as an opportunity to understand their disease and have a focus on lifestyle management of the condition, some found this an unwelcome reminder. Individuals with more advanced PKD, particularly those who had received a kidney transplant, reported feeling that they identified less as someone with PKD, and felt that the programme may be more appropriate for those at earlier stages of diagnosis, and could therefore be utilized as an introduction to disease management. A large qualitative study has demonstrated that both clinicians and people living with ADPKD felt that early support is required in order to manage psychological distress and address the level of uncertainty that people face, as well as provide education and tailored information [4]. It may therefore be important to consider at what stage this PKD-specific DHI is offered within the care pathway.

To date, limited research has been undertaken evaluating the role of physical activity interventions for people with PKD. It is understood from the literature that individuals with ADPKD have impaired physical capacity, as measured by maximal (peak oxygen uptake; VO_2_ peak) and submaximal indices of aerobic fitness in comparison with the general population [11]. Whilst results from the PKD-specific substudy did not reveal statistically significant improvements in physical function, as measured by the STS-60, there was a significant reduction in BMI, suggesting a potential weight management benefit. Qualitative analyses did not focus on the physical activity content of the platform, as this has been explored through previous research in the main Kidney BEAM trial, which had already demonstrated the ability of the Kidney BEAM platform to support individuals to engage with physical activity interventions [16]. Future studies might consider adapting kidney beam to provide bespoke training in other individual kidney diseases where need exists, e.g. diabetics with peripheral sensory loss or amputations.

### Strengths and limitations

This work aimed to understand the role of a PKD-specific education and physical activity DHI on the mental HRQoL in adults living with PKD. To date, limited research in this field has focussed specifically on individuals with PKD, and so it is a strength of this substudy that a 12-week physical activity and educational programme has been able to demonstrate improvements in HRQoL and other important health outcome measures. Due to limited exclusion criteria, a wide range of individuals were included in the trial, making this research widely applicable to the overall PKD population. There was, however, a larger proportion of females than male participants, and also a lack of black participants recruited to the trial. We further acknowledge that there were few participants with comorbid diabetic and ischaemic heart disease, which limits generalization of the results for participants with comorbid conditions. Future studies should ensure that there is good representation of all ethnicities, to ensure that the results are applicable to the whole PKD population. The use of a mixed-methods approach provided a rich dataset that allowed for exploration of the use of the PKD Kidney BEAM programme as an acceptable solution for people living with PKD. Quantitative and qualitative data sets were collected and analysed separately and concurrently, before being integrated within a comprehensive mixed methods analysis. This ensured equal importance of both datasets. Qualitative reflexivity and rigor were achieved through reflexive diaries as well as collaborative working within both the qualitative team and the wider trial team. Due to the nature of the intervention, individuals in the intervention group of the study were not able to be blinded the intervention. Primary and some secondary outcome measures were self-reported which may have introduced bias. As a substudy of the Kidney BEAM trial [16], this substudy was not designed to have sufficient participants for specified power to detect given effect sizes and thus changes in outcomes must be interpreted with care.

## CONCLUSION

A PKD-specific DHI has the potential to improve mental HRQoL, self-management behaviour, and the ability to foster a sense of community and peer support for people with PKD. The results may support further implementation of physical activity interventions for individuals living with PKD, and further research should focus on when in the care pathway this type of intervention would be best delivered to support self-management behaviour for people with PKD.

## Supplementary Material

sfaf041_Supplemental_File

## Data Availability

The data underlying this article will be shared on reasonable request to the corresponding author.

## References

[1] Liebau MC, Mekahli D, Perrone R et al. Polycystic kidney disease drug development: a conference report. Kidney Med 2023;5:100596. 10.1016/j.xkme.2022.100596 36698747 PMC9867973

[2] Chapman AB, Devuyst O, Eckardt K-U et al. Autosomal-dominant polycystic kidney disease (ADPKD): executive summary from a Kidney Disease: Improving Global Outcomes (KDIGO) Controversies Conference. Kidney Int 2015;88:17–27. 10.1038/ki.2015.59 25786098 PMC4913350

[3] Spithoven EM, Kramer A, Meijer E et al. Renal replacement therapy for autosomal dominant polycystic kidney disease (ADPKD) in Europe: prevalence and survival—an analysis of data from the ERA-EDTA Registry. Nephrol Dial Transplant 2014;29:iv15–25. 10.1093/ndt/gfu017 25165182 PMC7611099

[4] Baker A, King D, Marsh J et al. Understanding the physical and emotional impact of early-stage ADPKD: experiences and perspectives of patients and physicians. Clin Kidney J 2015;8:531–7. 10.1093/ckj/sfv060 26413277 PMC4581379

[5] Simms RJ, Thong KM, Dworschak GC et al. Increased psychosocial risk, depression and reduced quality of life living with autosomal dominant polycystic kidney disease. Nephrol Dial Transplant 2016;31:1130–40. 10.1093/ndt/gfv299 26268712

[6] Hoover E, Holliday V, Merullo N et al. Pain and health-related quality of life in autosomal dominant polycystic kidney disease: results from a national patient-powered registry. Kidney Med 2024;6:100813. 10.1016/j.xkme.2024.100813 38689835 PMC11059322

[7] Steele CN, Nowak KL. Nonpharmacological management of autosomal dominant polycystic kidney disease. Adv Kidney Dis Health 2023;30:220–7. 10.1053/j.akdh.2022.12.008 37088524 PMC10353837

[8] Wilkinson TJ, Clarke AL, Nixon DGD et al. Prevalence and correlates of physical activity across kidney disease stages: an observational multicentre study. Nephrol Dial Transplant 2021;36:641–9. 10.1093/ndt/gfz235 31725147

[9] Roshanravan B, Robinson-Cohen C, Patel KV et al. Association between physical performance and all-cause mortality in CKD. J Am Soc Nephrol 2013;24:822–30. 10.1681/ASN.2012070702 23599380 PMC3636794

[10] Capelli I, Lerario S, Aiello V et al. Diet and physical activity in adult dominant polycystic kidney disease: a review of the literature. Nutrients 2023;15. 10.3390/nu15112621 PMC10255338 37299584

[11] Reinecke NL, Cunha TM, Heilberg IP et al. Exercise capacity in polycystic kidney disease. Am J Kidney Dis 2014;64:239–46. 10.1053/j.ajkd.2014.03.014 24787761

[12] Lai S, Mastroluca D, Matino S et al. Early markers of cardiovascular risk in autosomal dominant polycystic kidney disease. Kidney Blood Press Res 2017;42:1290–302. 10.1159/000486011 29262409

[13] Martinez-Vea A, Bardaj A, Gutierrez C et al. Exercise blood pressure, cardiac structure, and diastolic function in young normotensive patients with polycystic kidney disease: a prehypertensive state. Am J Kidney Dis 2004;44:216–23. 10.1053/j.ajkd.2004.04.026 15264179

[14] Qiu J, Sato Y, Xu L et al. Chronic exercise protects against the progression of renal cyst growth and dysfunction in rats with polycystic kidney disease. Med Sci Sports Exerc 2021;53:2485–94. 10.1249/MSS.0000000000002737 34310502 PMC8594502

[15] NHS . NHS Long Term Plan 2019 [cited 24 September 2024]. Available from: https://www.longtermplan.nhs.uk/online-version/

[16] Greenwood SA, Young HML, Briggs J et al. Evaluating the effect of a digital health intervention to enhance physical activity in people with chronic kidney disease (Kidney BEAM): a multicentre, randomised controlled trial in the UK. Lancet Digit Health 2024;6:e23–32. 10.1016/S2589-7500(23)00204-2 37968170

[17] Greenwood SA, Briggs J, Walklin C et al. Kidney Beam-a cost-effective digital intervention to improve mental health. Kidney Int Rep 2024;9:3204–17. 10.1016/j.ekir.2024.08.030 39534205 PMC11551101

[18] Greenwood SA, Koufaki P, Rush R et al. Exercise counselling practices for patients with chronic kidney disease in the UK: a renal multidisciplinary team perspective. Nephron Clin Pract 2014;128:67–72. 10.1159/000363453 25358965

[19] UKKA . A multi-professional renal workforce plan for adults and children with kidney disease 2020 [cited 2024 23 September 2024]. Available from: https://ukkidney.org/sites/renal.org/files/FINAL-WFP-OCT-2020_compressed.pdf (19 December 2024, date last accessed).

[20] Walklin CG, Young HML, Asghari E et al. The effect of a novel, digital physical activity and emotional well-being intervention on health-related quality of life in people with chronic kidney disease: trial design and baseline data from a multicentre prospective, wait-list randomised controlled trial (kidney BEAM). BMC Nephrol 2023;24:122. 37131125 10.1186/s12882-023-03173-7 PMC10152439

[21] Lightfoot CJ, Wilkinson TJ, Memory KE et al. Reliability and validity of the patient activation measure in kidney disease: results of Rasch analysis. Clin J Am Soc Nephrol 2021;16:880–8. 10.2215/CJN.19611220 34117081 PMC8216620

[22] MacRae JM, Harasemiw O, Lightfoot CJ et al. Measurement properties of performance-based measures to assess physical function in chronic kidney disease: recommendations from a COSMIN systematic review. Clin Kidney J 2023;16:2108–28. 10.1093/ckj/sfad170 37915888 PMC10616478

[23] Braun V, Clarke V. Reflecting on reflexive thematic analysis. Qual Res Sport Exerc Health 2019;11:589–97. 10.1080/2159676X.2019.1628806

[24] Tong A, Sainsbury P, Craig J. Consolidated criteria for reporting qualitative research (COREQ): a 32-item checklist for interviews and focus groups. Int J Qual Health Care 2007;19:349–57. 10.1093/intqhc/mzm042 17872937

[25] Tong A, Rangan GK, Ruospo M et al. A painful inheritance-patient perspectives on living with polycystic kidney disease: thematic synthesis of qualitative research. Nephrol Dial Transplant 2015;30:790–800. 10.1093/ndt/gfv010 25637642

[26] Baker A, King D, Marsh J et al. Understanding the physical and emotional impact of early-stage ADPKD: experiences and perspectives of patients and physicians. Clin Kidney J 2015;8:531–7. 10.1093/ckj/sfv060 26413277 PMC4581379

[27] Eriksson D, Karlsson L, Eklund O et al. Health-related quality of life across all stages of autosomal dominant polycystic kidney disease. Nephrol Dial Transplant 2017;32:2106–11. 27662885 10.1093/ndt/gfw335 PMC5837636

[28] Miskulin DC, Abebe KZ, Chapman AB et al. Health-related quality of life in patients with autosomal dominant polycystic kidney disease and CKD stages 1-4: a cross-sectional study. Am J Kidney Dis 2014;63:214–26. 10.1053/j.ajkd.2013.08.017 24183837 PMC4075014

[29] Sanon Aigbogun M, Oberdhan D, Doane MJ et al. Disconnect in assessments of autosomal dominant polycystic kidney disease burden between patients and physicians: a survey study. Int J Nephrol Renovasc Dis 2021;14:105–15. 10.2147/IJNRD.S297491 33880055 PMC8053527

[30] Pérez-Dominguez T, Rodríguez-Pérez A, García-Bello MA et al. Progression of chronic kidney disease. Prevalence of anxiety and depression in autosomal dominant polycystic kidney disease. Nefrologia 2012;32:397–9. 22592426 10.3265/Nefrologia.pre2012.Feb.11379

[31] Lightfoot CJ, Nair D, Bennett PN et al. Patient activation: the cornerstone of effective self-management in chronic kidney disease? Kidney Dial 2022;2:91–105. 10.3390/kidneydial2010012 37101653 PMC10127536

[32] Hibbard JH, Greene J. What the evidence shows about patient activation: better health outcomes and care experiences; fewer data on costs. Health Aff (Millwood) 2013;32:207–14. 10.1377/hlthaff.2012.1061 23381511

