# Supplementary material for: Digital physical activity intervention via the Kidney BEAM platform in patients with polycystic kidney disease: a randomized controlled trial
Source: Clin Kidney J. 2025 Feb 12;18(3):sfaf041. doi: 10.1093/ckj/sfaf041 (PMC11892433; doi:10.1093/ckj/sfaf041)
Supplement: sfaf041_Supplemental_File [file sfaf041_supplemental_file.docx]

**Supplementary Material**

*Supplementary material 1.0 Per-protocol analyses*

| **Outcome measure** | **n** | **Baseline** | **12 weeks** | **Mean difference in change between groups (Kidney BEAM - waitlist control)** | **p value** |
| --- | --- | --- | --- | --- | --- |
|  |  | mean (SD) | mean (SD) | mean 95% CI |  |
| **Primary outcome** | | | | | |
| **KDQoL MCS (AU)** |  |  |  |  |  |
| Kidney BEAM | 23 | 47.86 (8.73) | 52.73 (7.68) | 5.87 (2.40-9.34) | .001 |
| Waitlist control | 28 | 48.08 (10.21) | 46.52 (9.47) |  |  |
| **Secondary outcomes** | | | | | |
| **KDQOL PCS (AU)** |  |  |  |  |  |
| Kidney BEAM | 23 | 45.70 (11.27) | 45.08 (11.38) | -1.05 (-5.30-3.19) | .621 |
| Waitlist control | 28 | 43.12 (11.73) | 44.23 (13.03) |  |  |
| **Symptom problem list** |  |  |  |  |  |
| Kidney BEAM | 21 | 83.76 (13.21) | 84.40 (12.90) | -0.73 (-4.89-3.43) | .726 |
| Waitlist control | 28 | 81.59 (17.17) | 82.90 (16.67) |  |  |
| **Effects of Kidney Disease** |  |  |  |  |  |
| Kidney BEAM | 23 | 80.47 (19.98) | 76.30 (23.02) | -2.41 (-13.9-9.12) | .676 |
| Waitlist control | 28 | 83.48 (15.54) | 81.36 (22.96) |  |  |
| **Burden of Kidney Disease** |  |  |  |  |  |
| Kidney BEAM | 23 | 64.32 (28.46) | 72.66 (21.87) | 4.10 (1.72-18.23) | .019 |
| Waitlist control | 28 | 73.21 (23.68) | 69.64 (26.45) |  |  |
| **Work status** |  |  |  |  |  |
| Kidney BEAM | 23 | 77.08 (36.05) | 77.08 (32.9 | -3.79 (-16.18-8.59) | .541 |
| Waitlist control | 28 | 80.36 (34.26) | 83.93 (33.48) |  |  |
| **Cognitive function** |  |  |  |  |  |
| Kidney BEAM | 23 | 79.72 (17.10) | 88.05 (10.21) | 8.06 (1.23-14.89) | .022 |
| Waitlist control | 28 | 78.09 (17.67) | 79.29 (21.65) |  |  |
| **Quality of social interaction** |  |  |  |  |  |
| Kidney BEAM | 23 | 77.22 (16.90) | 79.72 (12.51) | 0.11 (-7.91-8.13) | .978 |
| Waitlist control | 28 | 73.81 (16.22) | 77.86 (19.80) |  |  |
| **Sexual function** |  |  |  |  |  |
| Kidney BEAM | 6 | 45.83 (45.87) | 50.00 (44.72) | 13.93 (-45.80-73.66) | .621 |
| Waitlist control | 10 | 42.50 (47.21) | 55.00 (48.66) |  |  |
| **Sleep** |  |  |  |  |  |
| Kidney BEAM | 23 | 55.10 (16.46) | 58.44 (19.04 ) | 0.98 (-7.55-952) | .818 |
| Waitlist control | 28 | 64.11 (17.40) | 65.36 (21.20) |  |  |
| **Social support** |  |  |  |  |  |
| Kidney BEAM | 22 | 77.54 (3082) | 86.96 (18.10) | 13.69 (-0.76-28.14) | .063 |
| Waitlist control | 23 | 77.54 (24.42) | 74.64 (32.13) |  |  |
| **Dialysis staff encouragement** |  |  |  |  |  |
| Kidney BEAM | 3 | 83.33 (28.87) | 83.33 (28.87) | -4.67 (-61.12-51.77) | .756 |
| Waitlist control | 3 | 66.67 (28.87) | 75.00 (25.00) |  |  |
| **Overall health** |  |  |  |  |  |
| Kidney BEAM | 23 | 63.75 (19.29) | 65.83 (18.16) | -3.13 (-11.06-4.80) | .431 |
| Waitlist control | 28 | 60.71 (19.61) | 66.79 (21.44) |  |  |
| **Patient satisfaction** |  |  |  |  |  |
| Kidney BEAM | 3 | 83.33 (0.00) | 83.33 (16.67) | 8.45 (-43.00-59.89) | .672 |
| Waitlist control | 5 | 80.00 (21.73) | 76.67 (25.28) |  |  |
| **Physical functioning** |  |  |  |  |  |
| Kidney BEAM | 23 | 77.50 (21.11) | 76.87 (22.16) | 0.71 (-10.61-12.04) | .900 |
| Waitlist control | 28 | 75.71 (28.95) | 74.82 (28.30) |  |  |
| **Role physical** |  |  |  |  |  |
| Kidney BEAM | 23 | 70.83 (38.07) | 72.92 (38.25) | 5.46 (10.05-0.59) | .589 |
| Waitlist control | 28 | 62.50 (43.83) | 64.29 (45.35) |  |  |
| **Pain** |  |  |  |  |  |
| Kidney BEAM | 23 | 70.52 (27.37) | 68.96 (23.95) | -2.78 (-14.41-8.85) | .633 |
| Waitlist control | 28 | 62.41 (28.73) | 65.98 (33.23) |  |  |
| **General health** |  |  |  |  |  |
| Kidney BEAM | 22 | 46.52 (20.86) | 45.43 (21.79) | -4.28 (-12.16-3.59) | .279 |
| Waitlist control | 28 | 42.14 (21.45) | 45.36 (23.72) |  |  |
| **Emotional wellbeing** |  |  |  |  |  |
| Kidney BEAM | 23 | 73.17 (15.62) | 81.33 (10.66) | 12.04 (5.92-18.17) | <.001 |
| Waitlist control | 28 | 73.71 (17.33) | 69.71 (18.20) |  |  |
| **Role emotional** |  |  |  |  |  |
| Kidney BEAM | 23 | 83.33 (32.60) | 83.33 (34.05) | -2.97 (-20.08-14.14) | .729 |
| Waitlist control | 28 | 75.00 (39.15) | 80.95 (33.24) |  |  |
| **Social function** |  |  |  |  |  |
| Kidney BEAM | 23 | 74.48 (20.68) | 82.81 (18.73) | 6.52 (-4.07-17.12) | .222 |
| Waitlist control | 28 | 65.18 (35.41) | 69.64 (32.35) |  |  |
| **Energy/fatigue** |  |  |  |  |  |
| Kidney BEAM | 23 | 41.67 (23.53) | 53.96 (23.95) | 11.79 (4.06-19.52) | .004 |
| Waitlist control | 28 | 43.21 (24.46) | 43.39 (24.80) |  |  |
| **EQ-5D-3L utility score** |  |  |  |  |  |
| Kidney BEAM | 23 | 0.80 (0.13) | 0.79 (0.19) | -0.01 (-0.09-0.06) | .769 |
| Waitlist control | 28 | 0.75 (0.26) | 0.76 (0.20) |  |  |
| **CFS** |  |  |  |  |  |
| Kidney BEAM | 9 | 2.56 (1.01) | 1.89 (0.78) | -0.21 (-0.84-0.42) | .498 |
| Waitlist control | 18 | 2.72 (0.67) | 2.17 (0.79) |  |  |
| **STS60** |  |  |  |  |  |
| Kidney BEAM | 8 | 28.63 (9.44) | 33.13 (11.61) | -0.21 (-5.30-4.87) | .931 |
| Waitlist control | 18 | 24.28 (6.71) | 28.28 (10.69) |  |  |
| **PAM-13** |  |  |  |  |  |
| Kidney BEAM | 25 | 62.78 (17.01) | 70.31 (16.22) | 8.81 (2.16-15.46) | .010 |
| Waitlist control | 28 | 68.47 (17.12) | 65.53 (17.72) |  |  |
| **PHQ-4** |  |  |  |  |  |
| Kidney BEAM | 22 | 2.00 (3.20) | 1.70 (2.79) | -0.33 (-1.66-1.00) | .622 |
| Waitlist control | 28 | 2.21 (2.97) | 2.32 (3.17) |  |  |
| **Work and Social Adjustment Scale (WSAS)** |  |  |  |  |  |
| Kidney BEAM | 21 | 7.27 (6.87) | 6.95 (7.05) | -0.71 (-4.22-2.82) | .691 |
| Waitlist control | 28 | 9.82 (10.84) | 9.54 (10.70) |  |  |
| **eGFR** |  |  |  |  |  |
| Kidney BEAM | 19 | 37.79 (24.95) | 37.53 (25.23) | 2.70 (0.10-5.30) | .043 |
| Waitlist control | 18 | 46.56 (22.92) | 43.28 (21.02) |  |  |
| **Hb** |  |  |  |  |  |
| Kidney BEAM | 16 | 130.31 (13.96) | 123.56 (22.41) | -5.97 (-16.66-4.72) | .265 |
| Waitlist control | 26 | 129.46 (20.40) | 129.27 (18.80 |  |  |
| **Body mass** |  |  |  |  |  |
| Kidney BEAM | 20 | 82.60 (15.75) | 83.97 (16.29) | 1.44 (0.17-2.72) | .027 |
| Waitlist control | 25 | 81.81 (17.23) | 81.72 (17.48) |  |  |

Supplementary Material 1.0- Per Protocol Analysis

Supplementary Material 2.0- Qualitative Interviews additional supporting quotes

| **Theme** | **Sub-theme** |  |  |
| --- | --- | --- | --- |
| 1. Individualised acceptance | 1.1 Individual attitudes |  | “I’ve been very lucky, I didn’t have mm the high blood pressure, which often goes along with it. My brother did, has been struggling with his blood pressure, he’s younger than me actually. And he’s been struggling with it since he knew about it, which he was in his 30s maybe even earlier when he discovered it…sadly, he’s further down the line in terms of his kidney function, his has taken a rather you know serious nosedive and he’s heading for dialysis now even though he’s 6 years younger than me, so you can’t imagine how I feel about that.” KB387  “I think you can probably tell I try to put it out of my mind most of the time, but it was perhaps an unwelcome reminder of where I might end up.” KB381 |
|  | 1.2 Identity and experience |  | “…see now I feel like I’m a person living with a transplanted kidney rather than polycystic kidneys” KB378  “I might not be the ideal subject really for this, cause I never thought of it as polycystic kidney disease, I just thought the kidney was failing you know. I know what it was and what was causing it but it was basically just kidney failure and there was a reason why” KB356 |
| 2. Influences of engagement | 2.1 PKD Community |  | "I just like interacting with people, you know you would log on a couple of minutes before the class started, you start to recognise all the people, they say hello to you, you know it’s a live experience.” KB387  “It’s interesting being in a group with other people who are also going through what you’re going through” KB387  “It’s that connection with people in the same position and that there’s something you can join that sort of thing.” KB378 |
|  | 2.2 Dynamic |  | “I mean with the information videos I watched them and then that was it. And now I’m talking about the exercise videos as that’s what I primarily use it for.” KB390 |
|  | 2.3 Severity and timing |  | “I felt that I perhaps wasn’t quite the target viewership. I felt that it was targeted more for people further down the journey than me. Or perhaps people who were approaching or post transplant.” KB381  “Yeah generally, as I said I feel that I perhaps wasn’t erm I felt it was aimed at people who were sadly a bit further down the journey then I am currently.kb381  You know some of the videos, where there is people sat down, and they’re saying if you can’t stand up and I was thinking ‘ooh my word’ I don’t fancy that” KB381  “I was surprised there was all that information out there actually. I wish I had that from day 1 when I was diagnosed, it would have been So helpful, so helpful to have had that and more information.” KB387 |
| 3.Complementary empowerment | 3.1 Filling in the gaps |  | “I feel that even as someone who has it, I could learn more, I really liked that section of the kidney beam because it had the education and the exercise and it was really helpful to know certain things that I thought I would have already known but actually I’m sure I knew some of it but it was helpful as a refresher in some ways, and some ways new knowledge” K388  “I think that gives you the knowledge cause I think sometimes when you go to these doctors’ appointments, of course they explain to you what it is but then after that it’s about treatment. And you don’t go back and revisit it and you can’t be like wait a minute what was that and you’re just getting treated now.” KB388  “…They’re busy people… they often don’t have the time to answer the small funny questions I might have” KB387  “Yeah there was one in particular that explained the disease quite well. It explained a bit about the… it touched up on the gene that I’ve got and then it explained how the cysts can impact function and how they can get large which obviously impacts the function over time and sort of you know that was interesting. I think it was explained better to me in the video than it was by my consultant if I’m honest.” KB 381  “I like to think I’ve been doing the right thing, and this just confirms it. It is positive reinforcement.” KB390  “Yeah, look because it’s NHS sanctioned, and I know it’s coming from the NHS then I trust it.” KB 390 |
|  | 3.2 Empowerment |  | “Yeah, I felt good. I felt like I had some exercise which is great. I felt motivated” KB388  “There’s no magic button, id do anything to try and stay well and stay healthy.” KB381  "When I started taking part it was cold, wet and horrible at times and not the sort of thing where you might go out, you know if the weather is better you might be able to get out and walk and do more things, so I think also its very helpful when you’re stuck inside for any reason, but through the winter it was very useful for me.” KB387  “I like to think I’ve been doing the right thing, and this just confirms it. It is positive reinforcement.” KB390 |

Supplementary Material 4.0- Qualitative Interview team details

|  | Sex | Occupation | Experience | Credentials |
| --- | --- | --- | --- | --- |
| ER | F | Health Psychologist | PhD level experience in qualitative research data collection and methodology. | MSc, PhD |
| JB | F | Clinical and research Physiotherapist | Masters level training in qualitative research and methodology.  Previous experience of qualitative research and data collection. | BSc, PGCert |

Additional details

- There were no previous relationships with participants prior to the study
- The participants knew that the interviewer was a researcher
- No one else was present apart from researcher and interviewer during the interviews
- All interview team created topic guides with PPI input
- The interviewer used reflexive diaries to ensure their standpoint as physiotherapists and researchers in the field did not dictate code development
- Main analysis and coding was completed by ER with review and discussion with JB
- Interviews were conducted with patient and interviewer 1:1, via telephone
- No repeat interviews were conducted
- Interviews lasted between 36-55 minutes (average 44 minutes)
- Interview transcripts were not sent to participants
